# Supporting parents to care for a child with neurodevelopmental disability: exploring parents’ perspectives and experiences of a health service

**DOI:** 10.1186/s12913-025-13899-9

**Published:** 2025-12-16

**Authors:** Lyndal Hickey, Hanh Tu Duc Nguyen, Anna Bornemisza, Ingrid Sutherland, Daisy Shepherd, Anna Lucia, Miriam Yates, Gordon Baikie

**Affiliations:** 1 Department of Social Work, University of Melbourne, Melbourne, Australia; 2 Department of Neurodevelopment and Disability, The Royal Children’s Hospital, Melbourne, Australia; 3 Department of Social Work, The Royal Children’s Hospital, Melbourne, Australia; 4 Neurodisability and Rehabilitation, Murdoch Children’s Research Institute, Melbourne, Australia; 5 Clinical Epidemiology & Biostatistics Unit, Department of Paediatrics, University of Melbourne, Melbourne, Australia; 6 Department of Paediatrics, University of Melbourne, Melbourne, Australia

**Keywords:** Neurodevelopmental disability, Parents, Family, Children, Health services, Health

## Abstract

**Background:**

Health services play an essential role in supporting children with neurodevelopmental disability (NDD) and their families. Previous research shows parents need information, practical assistance with navigating systems and services, and holistic family-centred support, when caring for a child with disability. This article examines parents’ perspectives and experiences of the information and support provided to them to assist their care of their child with NDD, at the Neurodevelopment and Disability Clinic in Melbourne, Australia.

**Methods:**

An exploratory, cross-sectional study was conducted with 90 parents attending the clinic, using a survey with multiple choice and open-ended questions. Participants indicated whether they received, and how satisfied they were with 12 types of information and support for caring for a child with NDD. Subsequently, they described aspects of information/support that were helpful, not helpful, or mixed. Data was analysed using descriptive quantitative analysis and qualitative content analysis.

**Results:**

Amongst the 12 types of supports measured, participants were most likely to have received education about the child’s diagnosis and condition, supports relating to understanding the child’s diagnosis/condition, managing the child’s care, and obtaining medication. Participants were least likely to have received information about supporting siblings of a child with disability, assistance with financial and community resources, and social support with other families. Participants particularly valued information about the child’s condition, development, and care needs; support with accessing and coordinating hospital and community-based services; and support for family members. The qualities of health professionals and their approaches to delivering information and support shaped parents’ perception and experience of the healthcare they receive.

**Conclusions:**

Findings highlight how areas for health service and system development: providing the right information at the right time; practical assistance with accessing supports and services; family-centred support; and the perceived qualities, knowledge, and expertise of health professionals. Health professionals play a crucial and multi-faceted role in supporting children and family life with NDD.

**Supplementary Information:**

The online version contains supplementary material available at 10.1186/s12913-025-13899-9.

## Background

Health services play an essential role in the lives of children with neurodevelopmental disability and their families [1]. Neurodevelopmental disability (NDD) refers to disability arising from neurodevelopmental conditions that affect the structure or development of the brain and/or neuromuscular system, with functional impacts on movement, cognition, hearing, vision, communication, emotion, and behaviour [2, 3]. While a specific diagnosis may or may not be identified, cerebral palsy, autism spectrum disorder, and genetic birth anomaly syndromes are often associated with this term [2]. Neurodevelopmental conditions and the associated care needs can evolve over time at varied levels of severity and complexity; co-morbidity (i.e., co-occurrence of multiple conditions) is also common [1]. Developmental, care, and medical complexities may mean increased engagement and long-term involvement with health services when compared to other populations of children and young people. Sustained, targeted support is often needed throughout childhood and adolescence to promote positive outcomes [1]. However, healthcare use amongst children with NDD and their families has not been fully explored in the existing evidence base [4] and further research is needed to understand parents’ use of health services for information and support that assists them to care for their child with NDD. In this study, the term ‘parents’ is used inclusively to refer to people who identify in a parenting role with the child.

Caring for a child with complex care needs can be highly demanding on parents and family members; and caring for a child with NDD is associated with particularly complex challenges that are both rewarding and stressful [4, 5]. Parents juggle the child’s medical, behavioural, and social needs while navigating complex health, social, and family systems [6, 7]. Families managing complex care may also experience disruptions in multiple aspects of daily life at home, school, and in the community, which contribute to experiences of family conflict [7]. Furthermore, to meet the child’s specific needs, families are typically involved with services and programs across multiple sectors [4, 8, 9]. Existing research shows that parents look to health professionals for information and support to care for their child with NDD at home and other settings; and there is continued effort to understand how health professionals can provide such information and support [7, 8, 10–12].

The current literature identifies the importance for information or knowledge among families caring for a child with NDD specifically, and with disability more broadly. Parents want to acquire health knowledge and expertise to assist them in making informed decisions about treatment and formal support, and in managing care at home [10, 13]. Research has identified the following knowledge needs amongst parents of a child with complex health care needs: (1) knowledge about the condition/illness/diagnosis; (2) available supports; (3) treatment; (4) everyday care of the child; (5) the future; (6) how to explain illness to others; (7) equipment; (8) organisational issues; and (9) the effect of the illness on the family, with the most commonly mentioned being knowledge needs about the child’s condition and treatment, followed by where to obtain assistance and support [13]. Parents expect to receive information from health professionals, and to receive guidance from health professionals to navigate multiple, complex, and fragmented information sources [10]. Studies in Australia, Canada, and the UK report that parents of children with disabilities, intellectual disability, and cerebral palsy want information to be provided in an accessible, jargon-free, current, comprehensive, accurate, and balanced manner [8, 11, 14]. Information and knowledge needs can also vary across time, with parents indicating a need for the right information at the right time, provided in manageable increments [8, 10, 11]. Thus, the challenge is not only in the topics and amount of information parents can access, but *how*,* when*,* and from whom* this information is being obtained.

Practical support with navigating services and systems is also important for families of children with NDD, addressing needs arising from interactions across health, education, disability, social and community services sectors [4, 8, 9]. When parents receive adequate formal support across a variety of service areas, they report having more positive views of their child’s wellbeing [9]. Parents are not always aware of all the services and supports available to them; this highlights the need for health professionals to provide timely and appropriate referrals across often fragmented sectors [4, 8]. Nevertheless, parents remain tasked with coordinating and managing multiple supports. This involves having to repeat their stories with every new service and navigating differences in perspective and any power imbalances between themselves and care providers [4, 9]. Highly involved support may be beneficial to promote consistent engagement with services and reduce caregiver stress, with examples being care coordination programs in Canada and the UK [4, 15].

The holistic needs of the family also need to be considered to support the child’s care, promote the child’s positive health and developmental outcomes, and improve the family’s quality of life [8, 14, 16, 17]. Caring for a child with disability and caring for a child with NDD has been found to have both positive and negative impacts on family members. For parents, these positive impacts include improved family relationships, increased personal strength and personal development, a new outlook or perspective on life, and increased spirituality and religiosity. Conversely, negative impacts include increased stress, mental health difficulties, marital difficulties, increased caregiving responsibilities, loss of occupational opportunities, financial strain, and social isolation [4, 14, 18–21]. For siblings of children with NDD, impacts have been observed on emotional and mental health experiences, relationship with the parents and perception of parental availability, social competence and pro-social behaviours, peer relationships, and overall quality of life [16, 22]. Thus, holistic supports need to consider the family members’ experiences and wellbeing in multiple areas — health, emotional, psychological, relational, social, occupational, and financial [8, 11, 16, 17]. Examples of holistic services and interventions include emotional support and encouragement for all family members, recommendations and referrals to family and sibling supports, and facilitation of parents’ connection to peer and community networks [8, 14, 16, 23]. These supports are in line with a family-centred approach, which contrasts child-centred models that focus on treating a child’s impairments [24]. Family-centred services address the child’s experiences within broader family and community’s contexts, empowering parents to build their self-confidence, establish connections and attend to their own wellbeing [24].

This study aims to examine parents’ perspectives and experiences of the information and support provided to them to assist their care of their child with NDD, at an Australian health service specialising in NDD. Findings will inform healthcare delivery and extend our understanding of how health services meet the healthcare and support needs of children with NDD and their families.

## Methods

### Study design

This study was part of a larger research project undertaken in 2022–2023 at the Neurodevelopment and Disability clinic (NDD clinic) at The Royal Children’s Hospital, Melbourne, Australia, to examine family experiences of caring for a child with NDD [25]. Using a descriptive, exploratory, cross-sectional study design, data was obtained from parents caring for a child with NDD and attending the clinic, using a paper-based or online survey.

This research was conducted in accordance with the National Health and Research Council’s (NHMRC) National Statement on Ethical Conduct in Human Research (2007 and all subsequent updates). Ethics approval was received from The Royal Children’s Hospital Human Research Ethics Committee (RCH HREC # 76113).

### Setting

Every year, the NDD clinic provides tertiary and quaternary care to approximately 1500 children with neurodevelopmental conditions and other developmental and long-term conditions across the state of Victoria. This includes children with severe medical co-morbidities and/or at the more severe end of the physical or cognitive disability spectrums. This statewide clinic is staffed by specialists in both disability care and general paediatrics, providing medical case management (partnering with the child’s paediatrician in the community) and direct medical care, nursing and allied health professionals. Services include assessment, management, consultation, liaison, outreach, early intervention, advocacy, education, and research. Care provided is long-term, with most children involved with the clinic from infancy to 18 years. The clinic also offers a parent welfare service, acknowledging the significant stress on families and parents caring for a child with disability.

### Procedure

As part of the larger research project, two surveys were simultaneously distributed to adults (18 years and over) who identify as being in a parenting role for children and young people with NDD, and receiving treatment from the clinic, between August 2022 and September 2023. A convenience sampling method was used, as described below. The first survey (Parent survey) focused on parents’ experiences of caring for a child with NDD, and the second survey (Clinic survey) focused on parents’ perceptions of the clinic’s role in providing health and disability information and support to assist their care of their child. The Parent survey collected demographic, family household, and child characteristics; however, the Clinic survey did not collect any demographic information to ensure participant confidentiality. There was no link between the Parent survey and Clinic survey. This paper reports on the findings of the Clinic survey; the findings of the Parent survey will be reported in a separate publication.

#### Online recruitment

Researchers identified eligible participants by screening the Electronic Medical Record (EMR) NDD clinic list two weeks in advance of the child’s clinic appointment. Participants who were registered on their child’s EMR patient portal received a message about the study prior to their child’s upcoming appointment and were provided with the option to either complete the survey online via the REDCap survey platform, or in-person at the clinic. Participants received two reminder messages via the portal if they had not returned the survey two and four weeks after providing informed consent.

#### In-person recruitment

Participants who were not registered on their child’s EMR patient portal were approached directly at the clinic by two research team members (L and AB). These team members were not NDD clinicians, to maintain the confidentiality of the consent process and minimise any perceived pressure on parents whose children attend the NDD clinic. A poster about the study was also displayed in the clinic while researchers were present. These participants were provided with hard copies of the survey with unique identifiers and asked to return the hard copies via post within two weeks. Participants received a follow-up phone call after two weeks, and a reminder via mail after four weeks of providing informed consent.

Hard copy survey responses were inputted into REDCap for data management. The REDCap survey and electronic data platform was hosted at the University of Melbourne and accessed by the researchers only.

Consent to participate declaration: informed consent (online and hard copy) was obtained from all individual participants included in the study.

### Survey design

This article reports on the findings of the Clinic survey. The Clinic survey was designed by the research team and focused on parents’ perception of and experience with the NDD clinic. In the first section, participants were asked about the types of information and support provided to them by the clinic to assist them to care for their child. No existing measures were identified which captured this data. Therefore, the research team specifically developed 12 items for this study. Two team members (G & IS) are NDD clinicians and hold specialist knowledge and experience of the information and support provided to families within the scope of the NDD clinic’s service. Participants were asked to indicate if they had received each type of information/support, and subsequently, provide a satisfaction rating for this type of information/support on a five-point Likert scale, or indicate ‘Not applicable’. In the second section, participants were asked to describe the specific activities of the clinic that they found helpful or not helpful (or mixed) in three open-ended questions. No parameters were given for open-ended responses (e.g., number of words), as to not constrain the depth of participants’ responses. The survey is provided in Supplementary File 1.

### Participants

Out of 123 parents who consented to the larger research project, a total of 90 participants were recruited for this present study and the Clinic survey. As the Clinic survey did not collect demographic information, and the Parent survey and the Clinic survey were not linked, it is not possible to describe the demographic characteristics of this study’s sample. This limitation will be discussed in later sections of this article.

Characteristics of the full sample (*n* = 123) provide some insight into the parents who attended the NDD clinic and participated in the larger research project. The majority (92%) identified as the mother of the child attending the clinic. Most of the participants (72.95%) were born in Australia and approximately a quarter (24.39%) spoke a language other than English at home. Most of the children of participants (95.9%) were born in Australia, with a mean age of approximately ten years. Among participants who reported their child’s condition, approximately a quarter (28.32%) reported cognitive impairment and a quarter (23.89%) reported cerebral palsy. Participants reported a large range of initial diagnoses which children had received, including common conditions (e.g. Cerebral Palsy, ASD, Down Syndrome, Spina Bifida) as well as rare neurodevelopmental conditions (e.g., Rett syndrome, Cri du Chat syndrome, MPPH syndrome, Miller-Dieker Syndrome, Phelan-McDermid Syndrome, Joubert Syndrome). Many of the children had multiple diagnoses which they had received at different timepoints, and some children had not received a formal diagnosis. Most children (60%) were diagnosed after birth, and for most children (90.9%) it had been more than two years since their initial diagnosis. A complete description of the full sample is available in Supplementary File 2.

Furthermore, an examination of the Clinic survey’s open-ended responses provided some insight into the sample. Some participants and their children were new to the clinic; some had been involved with the clinic for several years, and some had attended the clinic for most of their child’s life, with one participant reporting they had attended the clinic for 17 years. One participant highlighted that they had limited experience with the clinic as it was usually the role of their partner to attend with their child. Some participants reported they had only interacted with a paediatrician; others described multi-disciplinary and multi-speciality links and referrals. Participants therefore had different levels of familiarity and experience with the clinic and the hospital.

### Data analysis

Descriptive quantitative analysis of the Clinic survey was conducted on available case data using Microsoft Excel [26] describing the frequencies of the types of information/support received and satisfaction with each type of information/support. A small number of contradicting responses were identified (e.g., where the participant indicated having received information/support but then provided a ‘Not applicable’ response on their satisfaction rating) but were included in analysis. Two duplicate responses were identified, completed in quick succession and contained almost all the same responses including open-ended responses. The earlier response was excluded from analysis.

Qualitative content analysis was undertaken using NVivo for open-ended responses, provided by 72 participants, to describe and summarise the specific activities of the clinic service that were helpful, not helpful, or mixed, in assisting parents in caring for their child. Content analysis was informed by the Inductive Content Analysis [27, 28]. Two researchers (XXX) individually familiarised themselves with the data and undertook an initial round of coding. Initial codes were subsequently compared and merged for an initial coding scheme. In the subsequent rounds of coding, open-ended responses were repeatedly coded line by line, and categories and subcategories iteratively refined. Finally, categories and subcategories were synthesised, connected, and interpreted to produce an overall understanding of parents’ experience and perception of the information and support provided to them in the clinic to assist them to care for their child.

## Results

### Parents’ satisfaction with the type of information and support received

The types of information/support reported by participants to assist them to care for their child and satisfaction ratings are summarised in Table 1.

**Table 1:** Types of information and support received by parents and their satisfaction with the information and support provided

Type of information and support	Received this support *n* (%)	Very unsatisfied/ Unsatisfied *n* (%)	Neutral *n* (%)	Satisfied/ Very satisfied *n* (%)
			*n*(%)	
Understanding your child’s diagnosis/condition	84 (93.33)	3 (3.33)	6 (6.67)	74 (82.22)
Adjustment to your child’s diagnosis/condition	75 (83.33)	5 (5.55)	12 (13.33)	69 (76.67)
Education about your child’s diagnosis/condition	85 (94.44)	6 (6.67)	11 (12.22)	67 (74.45)
Recognising your carer well-being needs	77 (85.56)	4 (4.44)	11 (12.22)	61 (67.78)
Understanding the impact of caring for your child	76 (84.44)	4 (4.44)	12 (13.33)	60 (66.67)
Meeting the needs of your family	73 (81.11)	3 (3.33)	14 (15.56)	56 (62.23)
Managing your child’s care	84 (93.33)	2 (2.22)	11 (12.22)	71 (78.89)
Obtaining medication for your child’s care	82 (91.11)	2 (2.22)	5 (5.56)	73 (81.11)
Information about supporting siblings of a child with disability	68 (75.56)	7 (7.77)	23 (25.56)	35 (38.89)
Understanding the hospital system	76 (84.44)	6 (6.66)	14 (15.56)	56 (62.22)
Assistance with resources such as finances and community services	64 (71.11)	13 (14.44)	14 (15.56)	33 (36.67)
Social support with other families in similar situation	57 (63.33)	12 (13.33)	23 (25.56)	16 (17.78)
Other	8 (8.89)	1 (1.11)	2 (2.22)	3 (3.33)

The most frequently received types of supports were education about the child’s diagnosis/condition; understanding the child’s diagnosis/condition; managing the child’s care and obtaining medication for the child’s care. The least frequently received types of support were information about supporting siblings of a child with disability; assistance with resources such as finances and community services, and social support with other families in similar situation. For almost all types of information and support, the majority of participants were satisfied or very satisfied. However, items with the highest dissatisfaction ratings were: supporting siblings of a child with disability; assistance with resources such as finances and community services; and social support with other families in similar situation. The highest numbers of neutral ratings were recorded for supporting siblings of a child with disability and social support with other families in similar situation.

Of the eight participants who indicated ‘Other’, four specified the types of information and support they received, which included support with getting a diagnosis, co-ordination with other clinics, referrals to external allied health services, and information and advice about medical conditions and treatment. Participants were satisfied with the support they received with getting a diagnosis and the information and advice provided about medical conditions and treatment, but dissatisfied about support with coordination with other clinics, and neutral about referrals to external allied health services.

### Parents’ perspective of the information and supports received

Open-ended responses were analysed to understand parents’ perspective of the information and support provided to them to assist them to care for their child. Five content categories were identified: (1) meeting information needs; (2) support with accessing, coordinating, and managing services; (3) support with caregiver, sibling, and family needs; (4) approach to delivering information and support; and (5) qualities of clinic staff and clinic interactions. The categories and subcategories are described in Table 2.

**Table 2:** Inductive content analysis – parents’ experiences of information and support received

Category	Sub-categories
1. Meeting information needs	· Information about child’s condition and care · Child’s development and how to support their development · Child and family needs at home and in the community
2. Support with accessing, coordinating and managing services	· Multidisciplinary and multi-specialty care support for complex care needs · Reassuring to have timely referrals and linkages to other departments for specialist support · Advocacy · Continuity of clinic support · Lack of coordinated appointments and communication between multi-specialist care services · Lack of knowledge about hospital system · Direct assistance to access funding support and systems beyond the hospital
3. Support with caregiver, sibling and family needs	• Family-centred approach • Family experiences of coping, care and hardships • Adjusting to diagnosis • Social supports for family members • Psycho-social wellbeing of family members
4. Approach to delivering information and support	• Holistic and strength-based • Positive, balanced outlook about the child’s condition was reassuring • Negative and challenging aspects about child’s condition was overwhelming • ‘When’ and ‘how’ information is delivered • Continuity of care with information and support provided consistently over time • Repeated contact with one health professional with knowledge of the child and family
5. Qualities of clinic staff and clinic interactions	• Empathy and understanding • Going ‘above and beyond’ routine care • Communication • Responsiveness in-between clinic appointments • Knowledge and expertise

#### Meeting information needs

The clinic played a key role in meeting the information needs of parents on a variety of different topics. Topics included: the child’s condition and care (i.e., the child’s diagnosis and condition, prognosis, pre-existing or co-occurring conditions, action/treatment/medical care plan, instructions on how to care for the child); the child’s development and how to support development; medication; equipment and available supports and services that may be helpful. There was also one reference to *“community and carer advice to us as parents/family*;” however, without further elaboration from the participant, it was not clear what specific information was provided. Regardless, this reference and references to the other topics of information provided, demonstrated that parents valued information pertaining to the child and family’s needs at home and in the community. Participants also found it useful to receive information consistently and frequently, particularly in the period after diagnosis. Repeated information was beneficial to reinforce existing knowledge and understanding.

Some participants expressed dissatisfaction with the lack of information they received about their child’s condition, care, and available supports. One participant referenced a lack of understanding of the child’s diagnosis, though it was unclear whether they were referring to their own or clinic staff’s lack of understanding. A lack of clarity around test results and how tests inform treatment contributed to another participant’s perception that clinic attendance was unnecessary:

*The scheduling of further tests that will have no real bearing on making life better or changing the treatment.*

Information about the condition therefore needed to be explicitly and meaningfully linked to care and treatment plans to be perceived as useful. In relation to available supports and services, some participants reported a lack of advice on the sibling, family, and financial supports available and a lack of awareness of the supports the clinic itself offered:

*Not understand all the services that are fully available to families with children with complex needs. Sometimes if we don’t bring up a topic*,* we would never know that service was available to us.*

Parents therefore highlighted the need to inform families of the supports available to them both internal and external to the health service.

#### Support with accessing, coordinating, and managing services

Beyond information, the clinic also provided more direct support to parents with accessing, coordinating, and managing services. The complex care of many children who attend the clinic meant that they required multidisciplinary and multi-specialty care; referrals were important for services not offered by the clinic. These included mental health and behavioural support, speech therapy, financial support, and other allied health supports, which may be provided by social workers, psychologists, speech pathologists, and other allied health specialists:

*Helping us access the support and care that we require that the NDD [clinic] cannot provide. Not able to provide services such as mental health and behavioural support.*

Timely referrals and linkages to other departments, multidisciplinary services, and specialist teams across the hospital contributed to a feeling of reassurance for parents:

*I can make contact via email with any concerns and know that someone will follow up with me or make referrals to the right teams.*

Advocacy was also crucial when assisting families to navigate multiple services:

*Chasing up other specialist teams for action. Advocate for our family when dealing with specialist teams.*

Continued support after the initial point of referral ensured the family was able to receive the care they needed across the hospital system. Some participants found navigating the hospital system to access these multi-disciplinary, multi-speciality services a significant concern. Participants experienced difficulties with working out how to make appointments and receiving timely appointment notifications. They also reported significant challenges with having multiple appointments across different days:

*Appointments for several services including other specialists*,* X-Rays/scans/MRIs often not coordinated. This requires several visits sometimes days or weeks apart. Travel time is over an hour. My grandson misses school on the day.*

*… it really does feel like we see five/six separate doctors at the hospital*,* not one “clinic”.*

The lack of coordinated appointments between the NDD clinic and other areas of the hospital contributed to experiences of treatment burden. Furthermore, issues were highlighted with the coordination and communication between multiple services and specialties:

*…our paediatrician isn’t made aware if/when we have a hospital (RCH) admission.*

*Communication sometimes between departments are mis-informed or mis-interpreted.*

There were some other concerns about the hospital system. One participant expressed a lack of knowledge on how the hospital system works, though it was unclear whether they were referring to their own or the clinic health professionals. Another response demonstrated a mismatch between the participant and hospital staff’s views about a child’s inpatient care needs:

*Unhappy about the system of hospital. With patients like my son*,* he should be given priority*,* I’ve been coming here for more than a year explaining he is crying*,* not sleeping*,* asking doctors to keep him in hospital for observation so they can help us better manage him. They never helped me. Never gave me a positive answer.*

One participant highlighted the need for a developmental perspective, supporting families to navigate the complex children’s hospital environment while also planning for transition to adult care. The participant suggested the need for social work services:

*We have never had anyone to guide us through this maze; we figured the hospital system out including parking…Especially being a teen and what to expect. Sometimes a social worker maybe helpful to. The next thing we know we will be on transition out of the [hospital].*

These responses demonstrated how participants located the clinic within the broader hospital system, and therefore, the need for a whole-of-hospital perspective when supporting families to access and coordinate the appropriate care for their child.

Participants also received direct assistance with accessing support and funding in other service systems beyond the hospital and healthcare setting. Participants reported that it was helpful when clinic staff assisted them with: paperwork and support letters for the National Disability Insurance Scheme (NDIS) — a national scheme which provides children and adults with disability individualised funding for disability-related equipment and supports in Australia [29]; Centrelink for social security income support; and the Companion Card scheme that supports their participation in the community and their child’s education. There was also one reference to helpful support with school and community inclusion. In contrast, some participants described a lack of connection between the clinic and broader service systems such as NDIS, Centrelink, and school systems, for example:

*It’s nice that we’re in the clinic*,* and I’m grateful*,* but there’s no clear support pathway or interaction with the NDIS or school system - or any real solutions.*

#### Support with parent, sibling, and family needs

The clinic’s family-centred approach involved supporting family members who care for the child. Participants described clinic staff who were interested in understanding, supporting, and addressing family experiences of coping, care, and hardships at home:

*Paediatrician very caring. Offers advice. Checks how we are all managing.*

*Dr [name] is very kind*,* empathetic in addressing not only medical needs but all aspects associated with the child and family life.*

Other helpful supports included adjustment support to diagnosis, social support for family members, and psychology services for parents. Conversely, some participants reported a lack of family support and a lack of support for adjustment to diagnosis. One participant expressed interest in sibling and social connection support, but was unsure about the clinic’s role in providing these supports:

*I didn’t think the clinic would have things for sibling support or social connection. Not sure if it would work? Maybe referrals to known services?*

#### Practice approaches to delivering information and support

The types of information and supports provided, and how they were delivered, contributed to families’ experiences and satisfaction with the clinic. Some participants described the importance of a holistic and strengths-based approach to delivering information and support. Clinic staff saw the child beyond their medical diagnosis:

*We only use NDD [clinic] for a paediatrician to ensure we have someone to help with other issues not related to diagnosis.*

The child was included in decisions about their own care:

*Everyone at the clinic involves my child in any major decisions. Also*,* my child has always been treated with care*,* respect and understanding. He feels listened to*,* understood*,* and involved.*

A holistic, strengths-based approach also included a balanced, positive outlook about the child’s condition. Participants appreciated clinic staff who provided reassurance and encouragement to the child and family and promoted an optimistic view about the future:

*We always leave Dr [name]’s appointments with a good plan and feeling really heard and optimistic about our son’s future.*

In contrast, an overfocus on the negative and challenging aspects could be overwhelming:

*Early on*,* too much focus on potential co-existing medical problems and overwhelming amount of information about how hard it all is.*

While information about co-existing conditions was useful, it was important to consider when and how it was most appropriate to deliver this information to promote a more balanced view of the child’s condition.

Participants also valued the continuity of care. Regular follow-up and review appointments with the clinic were seen as beneficial, to address changes to the child’s conditions and to ease the family’s worry about their child’s development. However, significant delays and wait times between appointments contributed to an inconsistent experience of health care:

*When we do see someone*,* they are helpful in adjusting things to help out child’s condition*,* but we aren’t there often enough. We were forgotten by the system.*

It was acknowledged by some participants that these difficulties were hospital-wide, suggesting a system-level issue rather than an issue with the clinic specifically. Another positive contribution to the continuity of care was the relationships participants built with clinic staff:

*Being able to develop a relationship with [the doctor] who gets to know you and your child well. I have found this incredibly valuable and comforting.*

Parents found it helpful to have repeated contact with one health professional they could continue working with throughout the child’s development.

#### Qualities of clinic staff and clinic interactions

The qualities and characteristics of clinic staff and the interactions participants had with the clinic contributed to their perception of the empathetic and supportive care delivered. Participants described clinic staff, practices, and interactions that were empathetic, genuine, kind, understanding, supportive, reassuring, respectful, helpful, caring, friendly, patient, and loving:

*We are very thankful for our amazing paediatrician [name]. [They are] patient*,* kind*,* caring and supportive. We would be lost without [them].*

Participants also recognised where clinic staff *“go above and beyond”* to provide support to children and their families. The quality of communication was also important, with participants reporting *“good*,*” “helpful*,*”* and *“honest”* communication from clinic staff, describing both themselves and their child as ”*feeling heard and listened to”*.

The clinic’s responsiveness and availability to provide information and support, and to address changes in the child’s conditions were important both during and between appointments. During appointments, while some of the clinic staff could appear to be “*rushed*,” others created opportunities and took their time to answer the family’s questions:

*Our main doctor…has been wonderful. Always takes extra time to go thoroughly through any questions or concerns I have.*

It was important to parents that the clinic exercised *“flexibility around offering support when needed most”.* Participants found comfort and reassurance in being able to get in touch in between appointments:

*They’re readily available when I have a question about my child’s condition.*

Others found it difficult to contact the clinic for questions and concerns, particularly when there were emerging issues that required a change in care or treatment:

*It’s hard to see/talk to someone if you have questions or need to adjust. Sometimes the matter is not that severe (urgent) to go through the emergency*,* but the [general practitioners] are useless in our case.*

One regionally based participant described how the absence of communication with health professionals between appointments contributed to feelings of isolation.

The level of knowledge and expertise of clinic staff was noted by parents as impacting on the perceived quality of the health information and resources provided. Some participants highlighted the valuable knowledge held by clinic staff, including medical and disability-specific knowledge:

*Holistic care provided by paediatrician who is knowledgeable about complex disabilities and very caring.*

Others highlighted concerns with less experienced health professionals:

*Lack of knowledge/resources. Feel like doctors are google doctors except the elderly ones.*

It is both knowledge and practice experience across a diverse range of presentations and complex health needs that participants looked for in health professionals.

## Discussion

This study examined parents’ experiences of the information and support provided to them in a Neurodevelopment and Disability clinic at a tertiary children’s hospital in Melbourne, Australia. Findings about the clinic have direct relevance and important implications for disability service provision in both local and international contexts. The holistic and long-term nature of the clinic is highlighted, with health specialists providing both disability care and general paediatrics across the continuum of care (inpatient, outpatient and community) to children with NDD and their family from infancy to 18 years.

Findings are consistent with the research evidence, stressing the importance of information about the child’s condition, development, and care needs; of assistance with accessing and coordinating care across multiple disciplines, services, and sectors; and of holistic family-centred support for family members’ psychosocial needs and circumstances [4, 8–11, 13–17, 23, 24, 30]. Quantitative results show that parents in this sample received a range of information and supports related to these needs at the clinic. Both quantitative and qualitative results demonstrate parents’ satisfaction with or positive evaluation of the information and supports they received. This study illuminates the valuable role of health professionals in providing information and support that assists families with caring for a child with NDD from infancy to adulthood as well as implications for furthering the examination and development of this role, as discussed below.

Findings suggest the importance of providing information to families attending the clinic about services and supports available across the hospital and in the community, but also about supports offered within the scope of the clinic itself. Parents’ need for information about available supports has been well established in the existing literature [4, 6, 10, 13]; however, what has not been highlighted is a need for communication about the caregiver, sibling, and family supports offered within the scope of the health service the family currently attends. The NDD clinic has recognised this need by including a parent welfare service which provides psychology and nursing support to parents; however, the extent to which this can further meet the support needs of all family members requires further exploration. Family supports may be particularly relevant to multidisciplinary clinics where multiple health professionals offer a wide range of interventions and supports; previous research suggests the different roles of various health professionals may not always be clear to parents [14]. However, we need to understand when and how information about the broad range of supports the clinic offers should be provided to help reduce parental stress and ensure that the families can access the right information at the right time [10]. Previous research has shown evidence that building knowledge about the child’s care is a cumulative process [7, 10, 15, 31]. Furthermore, the care needs of children with NDD evolve over time throughout childhood and adolescence, requiring responsive changes in the way that care is delivered [1]. Parents may experience significant uncertainty about whether the child is on track with their developmental milestones and what to do when developmental problems occur [31]. In addition, as many children experience co-morbidities and receive multiple diagnoses over time [1], parents require up-to-date condition-related information to continue providing effective care at every stage of development and diagnosis. Participants in this study pointed to the need for multiple opportunities to ask questions and receive information about caring for their child, both in and between the infrequent clinic appointments. This provided a level of comfort and reassurance in knowing that they could contact the clinic while awaiting their next in-person appointment, should questions arise. Our findings are also in line with previous research indicating that parents prefer to have a knowledgeable and accessible primary point of contact for support and information [7, 15, 31]. These findings speak to the uniqueness of the NDD clinic in providing long-term services to children with NDD and their families across the continuum of care and its role as a key point of contact for parents in this study.

Parents’ accounts also highlighted the need for and an expectation of practical assistance with accessing supports, services, and resources. This need for practical support has been previously identified in existing literature [11, 15, 23]. Specifically, research shows that parents expect health professionals to offer tangible and hands-on support rather than assuming a solely consultative role, and that health professionals can take “something off parents’ plates”, “lessen[ing] the load” for parents who have had to take on multiple, complex roles and tasks to care for their child with disability [11]. The findings from this study extend on our understanding of the types of supports and systems with which parents believe they need practical assistance within the Australian context. Participants mentioned receiving or needing assistance with accessing the NDIS, government payments and services, and education support for their child. It highlights the need for the NDD clinic and other disability and health services to be knowledgable about the changing policy landscape that is shaping the services and supports available to families who are caring for a child with NDD.

Strongly linked to this policy context, is the need for support with NDIS processes noted by the parents in this study. Children with NDD who attend this clinic are likely to be registered with the NDIS and parents experience of the scheme may shape their perceptions of caring for a child with disability. Ranasinghe et al. (2017) report that parents with children with developmental disability experience significant difficulties with the NDIS registration process due to a number of factors such as: the volume and complexity of application forms; lack of online information; parents’ limited computer skills; parents’ education level and socio-economic status; parents’ awareness of available supports; and limited information provided by health professionals [32]. Mothers of children with cerebral palsy have reported significant administrative challenges in understanding the NDIS system, explaining the condition and needs of their child, and managing repetitive administrative requirements [33]. Practical, hands-on assistance from health professionals with knowledge of the health and disability system interface may be necessary to ensure children with NDD and their families can access the NDIS-related funding and supports they need in Australia [34].

Questions remain as to how hands-on, practical support can be delivered within the limited scope of the NDD clinic and other health services, as well as how this support fits into the broader contexts of the health system. In this study, qualitative findings highlight parents’ mixed experiences with receiving hands-on support. Furthermore, quantitative findings showed that while overall levels of satisfaction with the clinic were high, participants expressed higher levels of dissatisfaction with assistance to accessing finances and community services when compared to other types of information and support that the clinic provides. It was not possible from to ascertain from the data the barriers to receiving these supports and the reasons for dissatisfaction amongst parents; this is an area for further examination. The NDD clinic is primarily delivered by paediatricians focused on the health needs of the child; providing social supports and education may be beyond the current service. Myers et al. (2024) evaluated an alternative model whereby this support was provided by care coordinators placed in the same service. Findings demonstrated that supports provided by care coordinators ease the pressure on health professionals (including allied health and social care staff), empower families, reduce parental burden, and increase families’ engagement with health services. Another consideration to explore is whether this practical support can be promoted through informal networks. Wong and Shorey (2022), in reviewing international literature on peer support for parents of children with NDD, find that peers can provide important support such as creating referrals and arrangements on behalf of parents, and go further to suggest that peer support be considered an adjunct support service by health providers. The inclusion of holistic care coordination approaches and the promotion of peer support may be a topic of further studies for the NDD clinic and similar provider contexts in Australia, with a consideration of time, resourcing, and professional capacity.

Our findings contribute to existing knowledge regarding the importance of family-centred care in neurodevelopmental disability health services. Parents in this study spoke of the need for social support, sibling support, psychological support for parents, support with adjusting to diagnosis, and more broadly, support for family life. Results suggest families had varied experiences of family and sibling support at the clinic, both in terms of receiving these types of supports and satisfaction with the supports provided. The barriers to receiving or providing family-centred supports were also not clear, making it an important area for further examination within the clinic. Previous research by Prest et al. (2024) highlights the challenges of delivering services that support the family within the constraints of publicly funded health in the UK. Further examination of these contexts in Australia and internationally will have important implications for how health services can provide holistic support that addresses the needs of the whole family.

This study also contributes to the existing body of literature by describing the importance parents place on the role of health professionals and the qualities that they value. In the context of NDD, discussion about the nature of the relationship between the family and health professionals has been limited. Parents in this study appreciated health professionals who delivered information and support in an empathetic, respectful, and honest manner. They valued professionals who were friendly, caring, and who patiently answered their questions. This is a considerable strength of the NDD clinic and likely reflects the relational aspect of the collaboration between families and health clinicians that develops over time. Similar to Myers et al. (2024) and Fortune et al. (2023), this study identified that trust between parents and health professionals appeared to be built over time with repeated contact, and families valued long-term involvement with the clinic over the course of their child’s development. Finally, this study also extends insight into how parents perceive the knowledge and expertise held by health professionals. Douglas et al. (2016) report that parents of children with intellectual disability are concerned about the lack of health professionals with disability expertise who can provide referrals to appropriate services. In this study, some concerns were similarly expressed about the knowledge and expertise of some doctors in the clinic. Moreover, findings suggest that age, confidence, and familiarity with similar cases were some criteria that parents used to evaluate the health professionals they work with, or factors which impacted their satisfaction with the relationship they had with these professionals. Future research may examine how to build trust and positive relationships between parents and health professionals, considering what parents expect and value from health professionals when attending NDD health services. Services may consider implications for education and upskilling, ensuring opportunities for continued professional development, as well as relational skills when providing long-term care to families with disability.

There are strengths and limitations to this study. The sample represents a relatively small number of families who attend the NDD clinic each year. However, it represents the majority (73%) of the participants who consented into the larger research project. Furthermore, the sample size of *n* = 90 is large when compared to existing qualitative studies on similar topics with much smaller sample sizes, varying from 10 to 20 participants [6–8, 10, 14, 15, 22, 30, 31]. Furthermore, unlike some NDD studies which focused on a limited range of conditions/diagnoses [6, 8, 10, 19, 22], this research project captured the perspectives of parents caring for children with a large range of different neurodevelopmental conditions and diagnoses. The larger, more diverse sample allowed for a valuable exploration of a range of perspectives and experiences; however, the data obtained is not able to focus on health interventions for specific diagnostic groups and levels of impairment. Considering the convenience sampling method used, and the relatively small sample size, this study makes no conclusions about the generalisability of the findings.

There are some limitations relating to the research design. To protect the confidentiality of participants, the Clinic survey did not collect any demographic information from its sample, nor was it linked to the Parent survey which collected demographic information from the larger sample. Not being able to describe the sample limits our understanding on the level of care provided to the children and how this impacted the support and information families need from the clinic. This study also reported on parents’ perceptions of the NDD clinic and how it supported their care of the child but did not ask specifically about parents’ expectations of the clinic in supporting care (though some participant responses provided insight into the supports they expected to receive from the clinic). This is an area of further examination for the NDD clinic and for other health services in Australia.

Finally, it was not possible to ascertain from the qualitative data obtained the clinical role of the health professionals providing parents with information and support. Follow-up interviews may be beneficial for a more in-depth understanding of the different types of information and supports that can be provided by different professionals. This is particularly important for multidisciplinary health services to consider.

## Conclusions

The findings underscore the important and valuable role of health services in providing information and support that assist parents to care for their child with NDD and address the holistic needs of the family. Findings highlight important areas to strengthen the clinic and health services more broadly: providing the right information at the right time; practical assistance with accessing supports and services; family-centred support; and the perceived qualities, knowledge, and expertise of health professionals. These areas must be considered within structural and professional contexts, including questions of time, resourcing, and staffing within a publicly funded health system, both in Australia and internationally.

## Supplementary Information

Below is the link to the electronic supplementary material.

Supplementary Material 1

Supplementary Material 2

## Data Availability

The participants of this study did not give written consent for their data to be shared publicly, so due to the sensitive nature of the research supporting data is not available.

## Abbreviations

NDD: Neurodevelopmental disability
NDD Clinic: Neurodevelopment and Disability Clinic
