# Supplementary material for: Supporting parents to care for a child with neurodevelopmental disability: exploring parents’ perspectives and experiences of a health service
Source: BMC Health Serv Res. 2025 Dec 16;26:95. doi: 10.1186/s12913-025-13899-9 (PMC12821309; doi:10.1186/s12913-025-13899-9)
Supplement: Supplementary file 1 — Supplementary Material 1 [file 12913_2025_13899_MOESM1_ESM.docx]

S1: Clinic Survey

| **What information and support has your child and family received at the NDD clinic *(tick all that apply)*** | 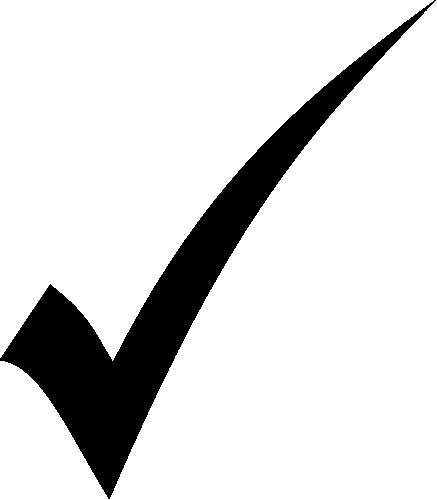 | **How would you rate your satisfaction with the NDD clinic information and support provided that assists you to care for your child?** | | | | | |
| --- | --- | --- | --- | --- | --- | --- | --- |
|  |  | Not applicable | Very unsatisfied | Unsatisfied | Neutral | Satisfied | Very Satisfied |
| Understanding your child’s diagnosis/condition |  | **⃞** | **⃞** | **⃞** | **⃞** | **⃞** | **⃞** |
| Adjustment to your child’s diagnosis/condition |  | **⃞** | **⃞** | **⃞** | **⃞** | **⃞** | **⃞** |
| Education about your child’s diagnosis/condition |  | **⃞** | **⃞** | **⃞** | **⃞** | **⃞** | **⃞** |
| Recognising your carer well-being needs |  | **⃞** | **⃞** | **⃞** | **⃞** | **⃞** | **⃞** |
| Understanding the impact of caring for your child |  | **⃞** | **⃞** | **⃞** | **⃞** | **⃞** | **⃞** |
| Meeting the needs of your family |  | **⃞** | **⃞** | **⃞** | **⃞** | **⃞** | **⃞** |
| Managing your child’s care |  | **⃞** | **⃞** | **⃞** | **⃞** | **⃞** | **⃞** |
| Obtaining medication for your child’s care |  | **⃞** | **⃞** | **⃞** | **⃞** | **⃞** | **⃞** |
| Information about supporting siblings of a child with disability |  | **⃞** | **⃞** | **⃞** | **⃞** | **⃞** | **⃞** |
| Understanding the hospital system |  | **⃞** | **⃞** | **⃞** | **⃞** | **⃞** | **⃞** |
| Assistance with resources such as finances and community services |  | **⃞** | **⃞** | **⃞** | **⃞** | **⃞** | **⃞** |
| Social Support with other families in similar situation |  | **⃞** | **⃞** | **⃞** | **⃞** | **⃞** | **⃞** |
| Other (please describe): |  | **⃞** | **⃞** | **⃞** | **⃞** | **⃞** | **⃞** |

| **What specific activities of the NDD clinic service are helpful?** |
| --- |
|  |
| **What specific activities of the NDD clinic service are not helpful?** |
|  |
| **If mixed, please describe?** |
|  |
