# Supplementary material for: Supporting parents to care for a child with neurodevelopmental disability: exploring parents’ perspectives and experiences of a health service
Source: BMC Health Serv Res. 2025 Dec 16;26:95. doi: 10.1186/s12913-025-13899-9 (PMC12821309; doi:10.1186/s12913-025-13899-9)
Supplement: Supplementary file 2 — Supplementary Material 2 [file 12913_2025_13899_MOESM2_ESM.docx]

**Table S1: Parent/Caregiver Demographics and Household Characteristics (n = 123)**

|  |  |  | Survey respondents  n (%) |
| --- | --- | --- | --- |

| **About parent/carer completing survey** | | | |  |  |
| --- | --- | --- | --- | --- | --- |
|  | Who completed the survey? – n (%) | | |  |  |
|  |  | Mother | | 101 (82.11) |  |
|  |  | Father | | 15 (12.20) |  |
|  |  | Grandparent | | 4 (3.25) |  |
|  |  | Aunty | | 1 (0.81) |  |
|  |  | Carer (not specified formal/informal) | | 1 (0.81) |  |
|  |  | Mother (not birth) | | 1 (0.81) |  |
|  | Country of birth^1^ – n (%) | | |  |  |
|  |  | Australia | | 89 (72.95) |  |
|  |  | Outside of Australia | | 33 (27.05) |  |
|  | Language other than English used at home: Yes – n (%) | | | 30 (24.39) |  |
|  | Highest level of education^2^ – n (%) | | |  |  |
|  |  | Less than high school | | 5 (4.13) |  |
|  |  | Graduated high school / VCE | | 11 (9.09) |  |
|  |  | Some university / TAFE | | 21 (17.36) |  |
|  |  | Graduated university / TAFE | | 37 (30.58) |  |
|  |  | Some post-graduate study | | 3 (2.48) |  |
|  |  | Post-graduate qualification | | 44 (36.36) |  |
|  | Part of faith-based or spiritual community: Yes^3^ – n (%) | | | 40 (33.61) |  |
| **About household** | | | |  |  |
|  | Who lives in the household – n (%) | | |  |  |
|  |  | Mother | | 108 (87.80) |  |
|  |  | Mother 2 | | 2 (1.63) |  |
|  |  | Father (including stepfather) | | 91 (72.36 + 1.63) |  |
|  |  | Extended family (including grandparents) | | 20 (4.88 + 11.38) |  |
|  |  | Carer (not specified formal/informal) | | 1 (0.81) |  |
|  |  | Siblings in household | |  |  |
|  |  |  | 0 | 33 (26.83) |  |
|  |  |  | 1 | 53 (43.09) |  |
|  |  |  | 2 | 28 (22.76) |  |
|  |  |  | 3+ | 9 (7.32) |  |
|  |  |  |  |  |  |
|  | | Someone pregnant or planning to adopt: Yes^2^ – n (%) | | | 3 (2.48) |
|  | | Parent’s/guardians’ relationship status^2^ – n (%) | | |  |
|  | |  | Married/partnered | | 100 (82.64) |
|  | |  | Separated/divorced | | 12 (9.92) |
|  | |  | Single | | 9 (7.44) |
|  |  |  |  |  |  |

^1^Missing values n = 1 (0.81%).
^2^Missing values n = 2 (1.63%).
^3^Missing values n = 4 (3.25%).
